# Metabolic compartmentalization between astroglia and neurons in physiological and pathophysiological conditions of the neurovascular unit

**DOI:** 10.1111/neup.12639

**Published:** 2020-02-09

**Authors:** Shinichi Takahashi

**Affiliations:** ^1^ Department of Neurology and Stroke Saitama Medical University International Medical Center Saitama Japan; ^2^ Department of Physiology Keio University School of Medicine Tokyo Japan

**Keywords:** astrocyte, D‐serine, ketone body, lactate, neurovascular unit (NVU)

## Abstract

Astroglia or astrocytes, the most abundant cells in the brain, are interposed between neuronal synapses and microvasculature in the brain gray matter. They play a pivotal role in brain metabolism as well as in the regulation of cerebral blood flow, taking advantage of their unique anatomical location. In particular, the astroglial cellular metabolic compartment exerts supportive roles in dedicating neurons to the generation of action potentials and protects them against oxidative stress associated with their high energy consumption. An impairment of normal astroglial function, therefore, can lead to numerous neurological disorders including stroke, neurodegenerative diseases, and neuroimmunological diseases, in which metabolic derangements accelerate neuronal damage. The neurovascular unit (NVU), the major components of which include neurons, microvessels, and astroglia, is a conceptual framework that was originally used to better understand the pathophysiology of cerebral ischemia. At present, the NVU is a tool for understanding normal brain physiology as well as the pathophysiology of numerous neurological disorders. The metabolic responses of astroglia in the NVU can be either protective or deleterious. This review focuses on three major metabolic compartments: (i) glucose and lactate; (ii) fatty acid and ketone bodies; and (iii) D‐ and L‐serine. Both the beneficial and the detrimental roles of compartmentalization between neurons and astroglia will be discussed. A better understanding of the astroglial metabolic response in the NVU is expected to lead to the development of novel therapeutic strategies for diverse neurological diseases.

## INTRODUCTION

Astroglia are one of the three types of glial cells in the brain: astroglia (astrocytes), oligodendroglia (oligodendrocytes), and microglia.^1^, ^2^, ^3^ Astroglia are the most abundant cells in the human brain and outnumber neurons by a factor of 1.4 in the human cerebral cortex.^4^, ^5^ In addition, their unique anatomical location, which is interposed between neurons and cerebral microvessels and was depicted more than 100 years ago in a sketch by a legendary neuropathologist, Santiago Ramón y Cajal, has been attracting the attention of many neuroscientists.^6^ In fact, neurons do not have any direct contact with microvessels despite their strict dependence on a continuous supply of glucose and oxygen from outside the brain through the cerebral blood flow. In contrast, 99% of the surfaces of brain capillaries are covered by astroglial foot processes (end‐feet), indicating that all essential materials supplied from the cerebral circulation must interact with astroglia before reaching the neurons.^7^ The other side of the astroglial end‐feet envelopes synapses in the brain cortex. Thus, synapses composed of presynaptic and postsynaptic neurons as well as astroglial end‐feet are known as tripartite synapses.^4^, ^5^, ^8^, ^9^ Moreover, astroglial cells are connected to each other via gap junctions with connexin 43 channels, forming a functional syncytium overall.^10^, ^11^, ^12^

The cardinal roles of astroglia in tripartite synapses are the maintenance of normal synaptic function through metabolic support by taking advantage of their anatomical location, which is interposed between synapses and microvessels.^13^, ^14^ Accumulating evidence supports the notion that astroglia are also key players in the regulation of cerebral blood flow.^15^, ^16^, ^17^, ^18^, ^19^, ^20^ The neurovascular unit (NVU) is a conceptual framework that was originally used to better understand the pathophysiology of cerebral ischemia.^21^, ^22^, ^23^ Now, the NVU is a tool that can be used to understand normal brain physiology as well as the pathophysiology of numerous neurological disorders.^24^, ^25^, ^26^, ^27^, ^28^, ^29^ Conversely, the malfunction of astroglia in the NVU (i.e., “astrogliopathy”)^30^, ^31^, ^32^, ^33^, ^34^ induces neuronal dysfunction, leading to various neurological disorders including cerebrovascular disease (e.g., stroke and small vessel disease‐like Binswanger's disease and cerebral autosomal‐dominant arteriopathy with subcortical infarct and leukoencephalopathy [CADASIL]/35 cerebral autosomal recessive arteriopathy with subcortical infarct and leukoencephalopathy [CARASIL]),^36^, ^37^ neurodegenerative disease (e.g., Alzheimer's disease,^38^, ^39^ Parkinson's disease,^40^, ^41^ and amyotrophic lateral sclerosis [ALS]),^42^, ^43^, ^44^ and neuroimmunological disease (e.g., multiple sclerosis [MS],^45^, ^46^, ^47^, ^48^ and neuromyelitis optica spectrum disorder [NMOSD]).^49^, ^50^ This review will focus on the supportive roles of astroglia in the NVU from the perspective of three major metabolic compartments with neurons: (i) glucose and lactate; (ii) fatty acid and ketone bodies (KBs); and (iii) D‐ and L‐serine.

## GLUCOSE AND LACATE

### Oxidative metabolism of glucose is mandatory for generating action potentials

The adult human brain weighs approximately 2% of the total body weight and consumes 25% of the total body glucose consumption and 20% of the oxygen consumption. More precisely, the cerebral metabolic rate of glucose consumption (CMR_glc_) and oxygen (CMRO_2_) in the adult brain is 31 pmol/100 g/min and 156 pmol/100 g/min, respectively.^51^, ^52^ The ratio of CMRO_2_/CMR_glc_ at resting state is approximately 5.5, which is close to 6.0, the theoretical stoichiometry for the complete oxidation of glucose with oxygen (C_6_H_12_O_6_ + 6O_2_ → 6CO_2_ + 6H_2_O). The measured data indicate that almost all glucose is metabolized oxidatively except for the consumption of some additional glucose for non‐oxidative metabolism. In fact, the brain is strictly dependent on glucose for its energy production.^51^, ^52^ High CMRO_2_ and CMR_glc_ levels produce ATP efficiently with a ratio of 6.0, driving Na^+^,K^+^‐ATPase to maintain a steep ionic gradient across the cellular membrane. When an action potential is generated, a rapid influx of Na^+^ and a slow efflux of K^+^ follow. The functional activation of the brain is locally related to glucose consumption, indicating that glucose utilization in the whole brain reflects mainly the neuronal consumption of glucose.

### Glucose utilization by astroglia

**Figure 1:**
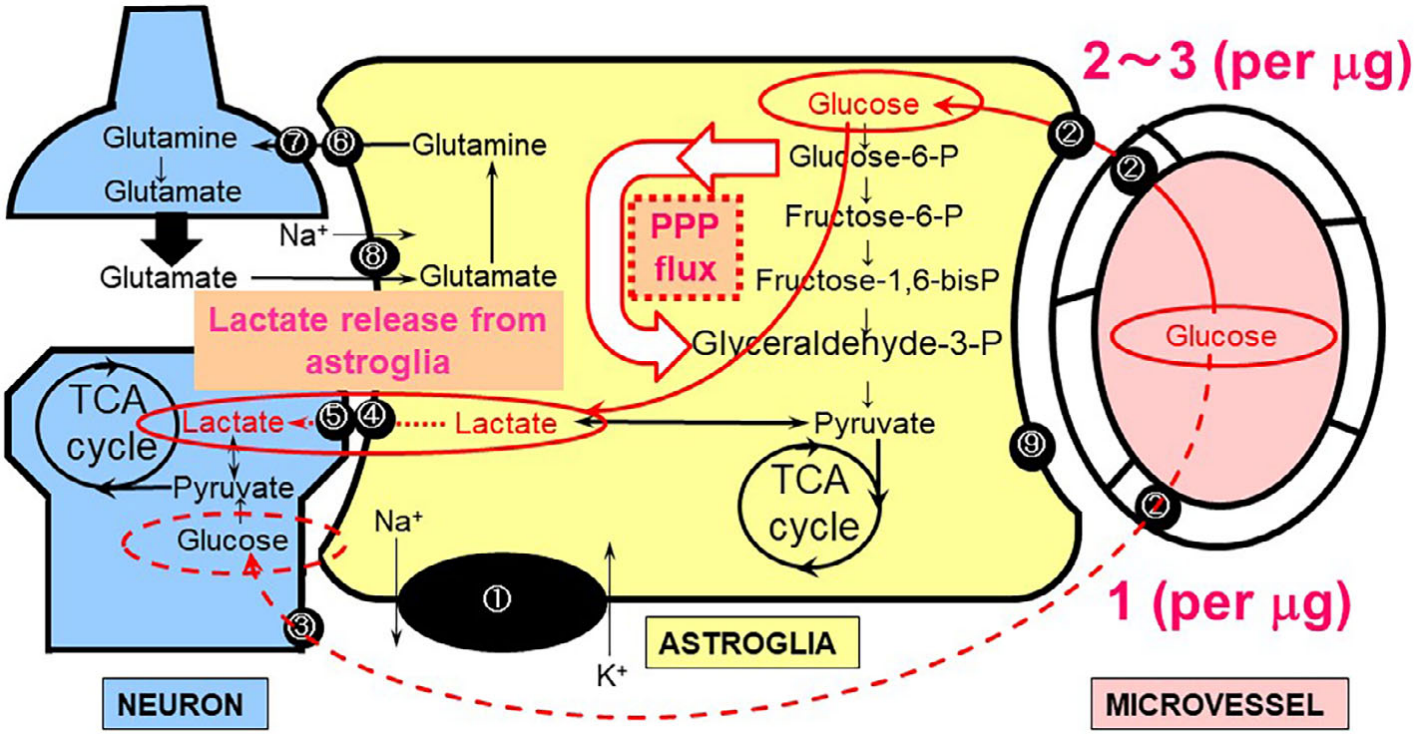
Metabolic compartment of glucose between astroglia and neurons in the neurovascular unit (NVU). Astroglia are interposed between microvessels and neuronal synapses, forming the NVU. Glucose supplied from outside the brain can be transported to and utilized by both astroglia and neurons (red lines). Glucose utilization by cultured astroglia (2–3 pmol/μg protein: red line) is two times higher than that in cultured neurons (1 pmol/μg protein: red broken line), and astroglia produce lactate even under normoxic conditions (aerobic glycolysis). Neuronal activation induces glutamate release from the pre‐synaptic nerve terminal; the glutamate is then taken up by astroglia, and this uptake, in turn, accelerates glucose consumption, leading to further lactate release. Lactate, then, serves as an energy substrate for neurons (astrocyte‐neuron lactate shuttle hypothesis, ANLSH). The basal pentose‐phosphate pathway (PPP) flux measured in cultured astroglia is approximately seven times higher than that in cultured neurons. Neuronal activation induces astroglial glycolysis, leading to increased flux to the PPP. Increases in NADPH in astroglia serve as a redox regulator that maintains the reduced form of glutathione. ① Na^+^,K^+^‐ATPase. ② Glucose transporter 1 (GLUT1). ③ Glucose transporter 3 (GLUT3). ④ Monocarboxylate transporter 1 (MCT1) and MCT4 (astrocytic form). ⑤ MCT2 (neuronal form). ⑥ System N transporter (astrocytic form). ⑦ System A transporter (neuronal form). ⑧ Na^+^‐dependent glutamate transporter‐1 (GLT‐1) and glutamate aspartate transporter (GLAST). ⑨ Fatty acid‐binding protein (FABP).

**Figure 2:**
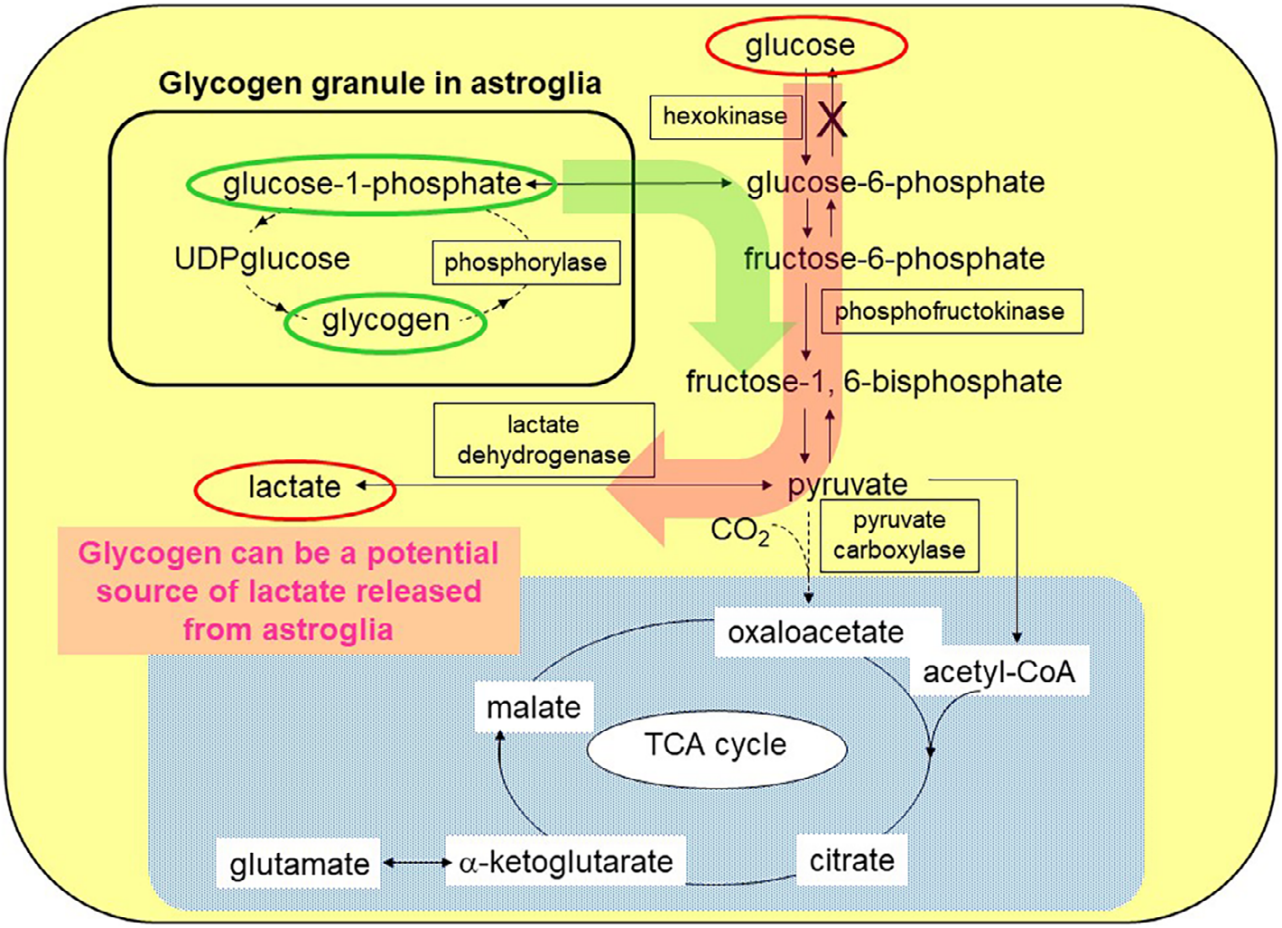
Glycogen deposits in astroglia are a potential source for lactate production. An astroglia‐specific enzyme, glycogen phosphorylase, degrades glycogen deposits in astroglia. In addition to glucose‐derived glucose‐6‐phosphate (pink arrow), glycogen‐derived glucose‐1‐phosphate (green arrow) is metabolized in a glycolytic pathway, producing lactate. When astroglia are cultured under high glucose conditions, the glycogen content and lactate production both increase.

Using fluorescently labeled glucose analogs, the cellular uptake of glucose can be evaluated in the brain cortex *in vivo*.^53^, ^54^ Glucose uptake occurs in neurons and astroglia via different glucose transporters (GLUTs), that is, GLUT3 and GLUT1, respectively (Fig. 1).^55^, ^56^ Since GLUT1 is also expressed in endothelial cells in brain capillaries, glucose supplied by the cerebral circulation can cross the blood–brain barrier (BBB). The congenital deficiency of GLUT1 induces intractable seizures beginning in infancy as well as mental retardation because of the unavailability of glucose to neural cells arising from limited glucose transportation through the endothelium.^57^, ^58^ Surprisingly, the brain has almost no storage of glucose, and glucose must be supplied continuously via the blood circulation.^53^, ^54^ More precisely, astroglia contain small amounts of glucose in the form of glycogen granules in their cell bodies.^59^ Glycogen is degraded by a glycogen phosphorylase (glycogenolysis), which is the astroglia‐specific enzyme, forming glucose‐1‐phosphate (G1P).^60^ G1P then enters the glycolytic metabolic pathway in astroglia (Fig. 2). A total amount of glycogen content measured in the brain can maintain its function for only 3 min, based on the CMR_glc_.^61^ If glucose derived from astroglial glycogen was available for neurons, it would be of some help. Unfortunately, G1P cannot cross the cell membrane because of its low lipid solubility. Instead, lactate or pyruvate, the end‐products of glycolysis, can exit astroglia via monocarboxylate transporter 1 (MCT1) and MCT4; they can then re‐enter neurons via monocarboxylate transporter 2 (MCT2), which is utilized as an energy source for neuronal tricarboxylic acid (TCA) cycle substrates (Fig. 1).^62^, ^63^, ^64^ Not only glycogen‐derived lactate/pyruvate, but also lactate/pyruvate from blood‐supplied glucose can be transferred to the neurons (Fig. 2).^65^ If this intercellular compartmentalization between neurons and astroglia operates in the resting and/or activated brain, the measured CMR_glc_ would reflect mainly astroglial glucose utilization, since neurons utilize lactate derived from astroglia. This hypothetical model, termed the “astrocyte‐neuron lactate shuttle hypothesis (ANLSH)”, was originally proposed by Pellerin and Magistretti in 1994 based on data obtained using cultured cells^66^ and was supported by our findings^67^, ^68^ but has remained controversial for more than a quarter of a century.^69^, ^70^

### Aerobic glycolysis in astroglia

Astroglia seem to be more strictly dependent on glucose, and their metabolism, at least in cultured astroglia *in vitro*, seems to be more glycolytic than that in cultured neurons.^71^ In fact, astroglia can survive if mitochondrial oxidative metabolism is inhibited, while neurons cannot.^72^ Glucose uptake or utilization (phosphorylation by hexokinase [HK]) can be quantified using cultured astroglia and neurons *in vitro* and *in vivo* using modern molecular techniques,^73^ while the quantitation of oxygen consumption is more difficult. However, importantly, the importance of the ANLSH is the capacity for lactate production by astroglia irrespective of an adequate supply of oxygen, that is, aerobic glycolysis. Although under cerebral ischemia, both astroglia and neurons produce large amounts of lactate that accumulate in tissues, experimental data show that astroglia *in vitro* produce a large amount of lactate under normal (21%) oxygen environments.^74^ In similar environments, cultured neurons produce less lactate from glucose. These phenomena have led to the hypothesis that glucose supplied from the cerebral circulation is taken up by mainly astroglia taking advantage of their anatomical location and is metabolized in astroglia to produce lactate, which is exported to neurons via MCT1 and 4. Neurons can technically utilize both glucose and lactate, as they express both GLUT3 and lactate transporter (MCT2). In contrast to GLUT1 deficiency, the congenital deficiency of neuron‐specific GLUT has not been reported. Only a mouse model of GLUT3‐knockout (KO) has been reported to exhibit autism‐like phenotypes, suggesting that neurons can survive without glucose under an environment where lactate is available.^75^ In fact, neuronal lactate dehydrogenase (LDH) isozyme favors the conversion of lactate to pyruvate, while astroglial LDH isozyme favors the formation of lactate from pyruvate.^76^ Supporting this notion, *in vitro* studies show that glucose consumption by neurons and that by astroglia are similar when lactate is not available. However, interestingly, when both glucose and lactate are available, cultured neurons preferentially utilize lactate as the TCA cycle substrate.^77^, ^78^, ^79^

**Figure 3:**
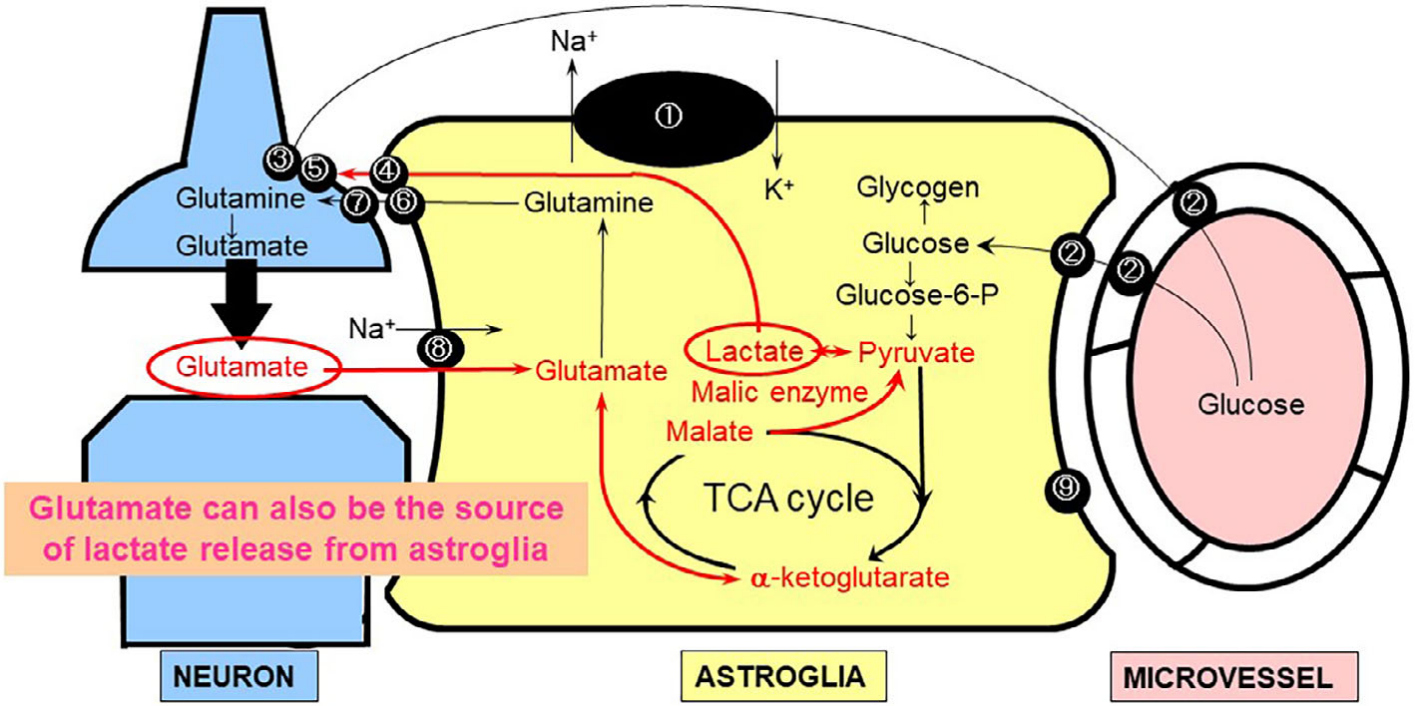
Glutamate taken up by astroglia serves as an astroglial tricarboxylic acid (TCA) cycle intermediate, leading to lactate production. Glutamate released from pre‐synaptic neurons is taken up by astroglia and recycled back to neurons (glutamate‐glutamine cycle). Some of the glutamate in astroglia is converted to α‐ketoglutarate, which enters the TCA cycle (red line) as an intermediate substrate, leading to astroglial lactate production by malic enzyme. ① Na^+^,K^+^‐ATPase. ② Glucose transporter 1 (GLUT1). ③ Glucose transporter 3 (GLUT3). ④ Monocarboxylate transporter 1 (MCT1) and MCT4 (astrocytic form). ⑤ MCT2 (neuronal form). ⑥ System N transporter (astrocytic form). ⑦ System A transporter (neuronal form). ⑧ Na^+^‐dependent glutamate transporter‐1 (GLT‐1) and glutamate aspartate transporter (GLAST). ⑨ Fatty acid‐binding protein (FABP)

The ANLSH emphasizes that astroglial lactate production is enhanced by glutamate stimulation in association with neuronal activation.^66^ Glutamate released from activated pre‐synaptic neurons is known to be rapidly taken up by astroglia that envelop the synapse.^80^, ^81^ The astroglial uptake of glutamate is dependent on the Na^+^‐dependent glutamate transporter‐1 (GLT‐1) and glutamate aspartate transporter (GLAST) (Fig. 1).^80^, ^81^ Co‐transported Na^+^ and glutamate increase the intracellular concentration of Na^+^ ([Na^+^]_i_), leading to the activation of Na^+^,K^+^‐ATPase; this, in turn, accelerates ATP production in astroglia. Whether ATP production in astroglia is dependent on glycolysis or the mitochondrial oxidative metabolism of glucose continues to be a matter of long‐lasting debate.^69^, ^70^, ^82^, ^83^ An *in vitro* experiment showed that glutamate stimulation does, indeed, increase glucose consumption and lactate production by astroglia (i.e., aerobic glycolysis).^66^, ^67^, ^68^, ^74^ The functional activation of *in vivo* brain also induces transient increases in lactate production locally in distinct lesions of an activated site where glucose utilization is eventually increased.^84^, ^85^ However, *in vivo* studies have not been able to determine the origin of the lactate because of a lack of cellular resolution. Even though astroglia produce lactate from glucose, astroglia are not necessarily devoid of mitochondrial function. Of note, glutamate that is taken up by astroglia can also serve as a substrate of the TCA cycle after its conversion to α‐ketoglutarate, implying that increased lactate production is not necessarily a reflection of solely glycolytic activation and that oxidative metabolism can also be activated using substrates other than glucose. Moreover, glutamate‐derived α‐ketoglutarate can also produce lactate during TCA cycle metabolism.^86^ Glutamate reportedly inhibits the neuronal utilization of glucose,^87^ implying a shift in the energy source from glucose to lactate (Fig. 3).

### Dual roles of lactate

Irrespective of accumulating evidence supporting the ANLSH, the fate of lactate, if it is really produced by astroglia via a glutamate signal from activated neurons, has remained a matter of controversy for more than 25 years.^69^, ^70^ The original ANLSH followed by numerous reports proposed that activated neurons consume lactate produced by astroglia.^66^, ^88^, ^89^, ^90^, ^91^, ^92^, ^93^, ^94^ A strong argument for this is based on a kinetic property of MCT2 expressed in neurons, which has a low kilometer value. The transportation of lactate into neurons becomes saturated at a low concentration of lactate, and neurons cannot utilize additional lactate even if it is produced by activated astroglia.^95^, ^96^, ^97^, ^98^, ^99^, ^100^, ^101^, ^102^, ^103^, ^104^, ^105^ Conversely, whether neuronal ATP production is exclusively dependent on the uptake of glucose by neurons remains uncertain. The *in vivo* truth remains to be clarified.

Another possible role of lactate is as a ligand of hydroxycarboxylic acid receptor 1 (HCAR1) expressed in neurons.^106^, ^107^ HCAR1, a G‐protein coupled receptor, suppresses neuronal excitability by increasing the intracellular cAMP concentration. Irrespective of the origin of lactate, direct evidence supports that extracellular lactate regulates synaptic activity (i.e., mainly inhibitory effects) and that the disinhibition of neuronal excitation can cause intractable epilepsy. Assuming that lactate is mainly produced by astroglia, the impairment of lactate production by astroglia can lead to neuronal damage through hyper‐excitation (i.e., excitotoxicity). In fact, neuronal degeneration in ALS, in which an unknown mechanism induces motor neuron death, has been hypothesized to be induced by the impairment of astroglial glutamate uptake as well as the hyper‐excitation of spinal motor neurons.^108^

Astroglial lactate production, especially that derived from glycogen (i.e., glycogenolysis), has been implicated in the formation of long‐term memory.^109^, ^110^ The exact mechanism by which lactate consolidates memories has not been elucidated and remains somewhat controversial.^103^, ^111^, ^112^ Several experiments support the idea that lactate produced in astroglia may serve as an energy source for neuronal synapse remodeling and gene expression, but not for the TCA cycle.^113^ The role of lactate as a ligand for HCAR1 remains to be elucidated.

### Neuroprotective roles of astroglial glycolysis and PPP

Of note, the functional activation of neurons increases glucose utilization locally, and both neurons and astroglia contribute to glucose consumption. We have focused on the roles of glucose metabolism, especially glycolysis in astroglia, from the perspective of their supportive aspects in the protection of neurons against oxidative stress.^30^, ^31^, ^32^, ^33^, ^34^ Neuronal energy production is dependent on mitochondrial oxidative metabolism, which produces small amounts of reactive oxygen species (ROS). Oxidative stress has been implicated in the pathogenesis of numerous neurological disorders (i.e., stroke, ALS, Parkinson's disease) as well as normal aging of the brain.^30^, ^31^, ^32^, ^33^, ^34^ The glutathione system acts as an intrinsic protective mechanism. Glutathione peroxidase reduces ROS by converting it to H_2_O_2_ in concert with the conversion of a reduced form of glutathione (GSH) to an oxidized form (GSSG).^30^, ^114^, ^115^ The maintenance of the GSH concentration is dependent on nicotinamide adenine dinucleotide phosphate (NADPH), a product of the pentose‐phosphate pathway (PPP), which is a shunt pathway of glycolysis (Fig. 1).^30^, ^51^, ^52^ Therefore, the influx to the PPP in glycolysis is an index of PPP activity and reflects the anti‐oxidative function of many kinds of cells. PPP flux is regulated by glucose‐6‐phosphate dehydrogenase (G6PDH), which is a rate‐limiting enzyme of this shunt pathway.^30^, ^51^, ^52^ The basal influx to the PPP in astroglia is approximately seven times as high as that in neurons, suggesting that glycolysis has anti‐oxidative roles in astroglia.^30^, ^31^

Increased glucose phosphorylation by HK leads to PPP flux through the allosteric regulation of G6PDH. Thus, a high glucose environment can activate PPP flux in astroglia.^30^, ^31^ In diabetic patients, a high plasma glucose concentration is associated with a high glucose content in the brain because of the facilitated diffusion of glucose by GLUT1. Whether a high glucose level in the brain readily increases glucose phosphorylation is debatable.^30^, ^31^ Because the Km (Michaelis constant) of HK is low, in clear contrast to glucokinase (GK) in the liver, glucose phosphorylation becomes saturated at a low glucose concentration.^51^, ^52^ The existence of high‐Km HK in the brain has been postulated. Our study showed that PPP flux increases according to the glucose concentration in an assay solution.^30^, ^31^ These observations might reflect the presence of high‐Km GK, like HK, in the brain. If this is true for *in vivo* brain, neuronal activation would induce astroglial glucose utilization via glutamate, leading to astroglial PPP activation. Importantly, the synthetic activity of glutathione, which consists of three amino acids, glutamine, cysteine, and alanine, is higher in astroglia than in neurons.^116^, ^117^ As mentioned above, the glycolytic activity as well as the PPP flux are dominant in astroglia, leading to the anti‐oxidative mechanism is astroglia.^30^, ^31^ When glutathione is synthesized, it must be transferred in a reduced form.^116^, ^117^ In addition, the reduced form of GSH itself can exert an anti‐oxidative role (see dopamine‐induced neurotoxicity).

### Transcriptional regulation of PPP flux by the Keap1/Nrf2 system

Another mechanism of PPP flux regulation is the transcriptional control of a key PPP enzyme. The rate‐limiting enzyme of PPP flux is G6PDH, and its transcription is under the control of the transcriptional factor nuclear factor‐erythroid‐2‐related factor 2 (Nrf2).^118^, ^119^ Nrf2 is anchored by an adaptor protein of Kelchlike ECH‐associated protein 1 (Keap1) in the cytosol. As a complex, Keap1/Nrf2 is degraded constantly by the proteasome system, and Nrf2 is not in an active state in terms of transcriptional control. When cells are exposed to various stress, a conformational modification of Keap1 or Nrf2 occurs and results in dissociated Nrf2 being released and binding to the antioxidant response element (ARE), where it initiates the transcription of anti‐stress response proteins including G6PDH.^118^, ^119^, ^120^, ^121^ Importantly, glutathione synthesis enzymes are also under the regulation of the Keap1/Nrf2 system. Astroglial glycolysis and its shunt pathway, the PPP, seem to play an important role in protecting neurons against oxidative stress through the Keap1/Nrf2 system.^30^, ^31^

Dopamine released from dopaminergic neurons is known to be auto‐oxidized to form dopamine quinone and ROS, which, in turn, damage neurons. Astroglia releases GSH and reduces dopamine‐induced ROS toxicity in Nrf2‐depenent manners. We have found that astroglia protect neurons against dopamine exposure by reducing ROS, while astroglia prepared from Nrf2‐KO mice are incapable of such activity.^34^ An *in vivo* model supports the notion that Nrf2‐KO mice are susceptible to a Parkinson's disease‐like phenotype and that an Nrf2 activator can modify the progression of Parkinson's disease.^34^

Neuro‐inflammation plays a cardinal role in the pathogenesis of stroke.^33^, ^122^, ^123^, ^124^ During the early phase of stroke, ischemic cell damage makes neurons release various kinds of molecules, such as damage‐associated molecular pattern, which, in turn, act as Toll‐like receptor 4 (TLR4) ligands. Microglia in which TLRs are expressed abundantly produce various pro‐inflammatory cytokines, accelerating neuronal damage. Nitric oxide (NO) is thought to be one of the molecules that plays a pro‐inflammatory role.^122^, ^123^, ^124^ Such actions of microglia switch astroglia from acting as neuroprotectors to acting as neurodamagers.^125^ However, we found that upon stimulation with lipopolysaccharide (LPS), a classical TLR ligand, microglia produce NO that diffuses into astroglia, activating the PPP through the S‐nitrosylation of Keap1, which facilitates Nrf2 translocation to the nucleus and triggers G6PDH transcriptional activation.^33^

## FATTY ACID AND KB

### KBs produced from fatty acids serve as energy substrates for neurons

**Figure 4:**
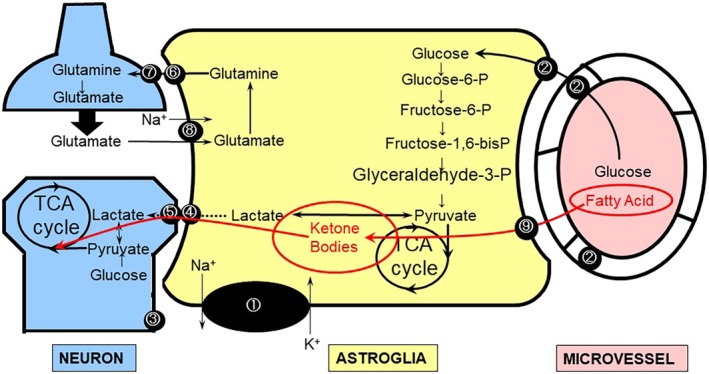
Ketone bodies (KBs) produced by astroglia serve as energy substrates for neurons. Fatty acids supplied from the blood are transported to astroglia in the brain, generating KBs; these KBs can fuel neurons as a tricarboxylic acid (TCA) cycle substrate (red line). ① Na^+^,K^+^‐ATPase. ② Glucose transporter 1 (GLUT1). ③ Glucose transporter 3 (GLUT3). ④ Monocarboxylate transporters 1 (MCT1) and MCT4 (astrocytic form). ⑤ MCT2 (neuronal form). ⑥ System N transporter (astrocytic form). ⑦ System A transporter (neuronal form). ⑧ Na^+^‐dependent glutamate transporter‐1 (GLT‐1) and glutamate aspartate transporter (GLAST). ⑨ Fatty acid‐binding protein (FABP).

**Figure 5:**
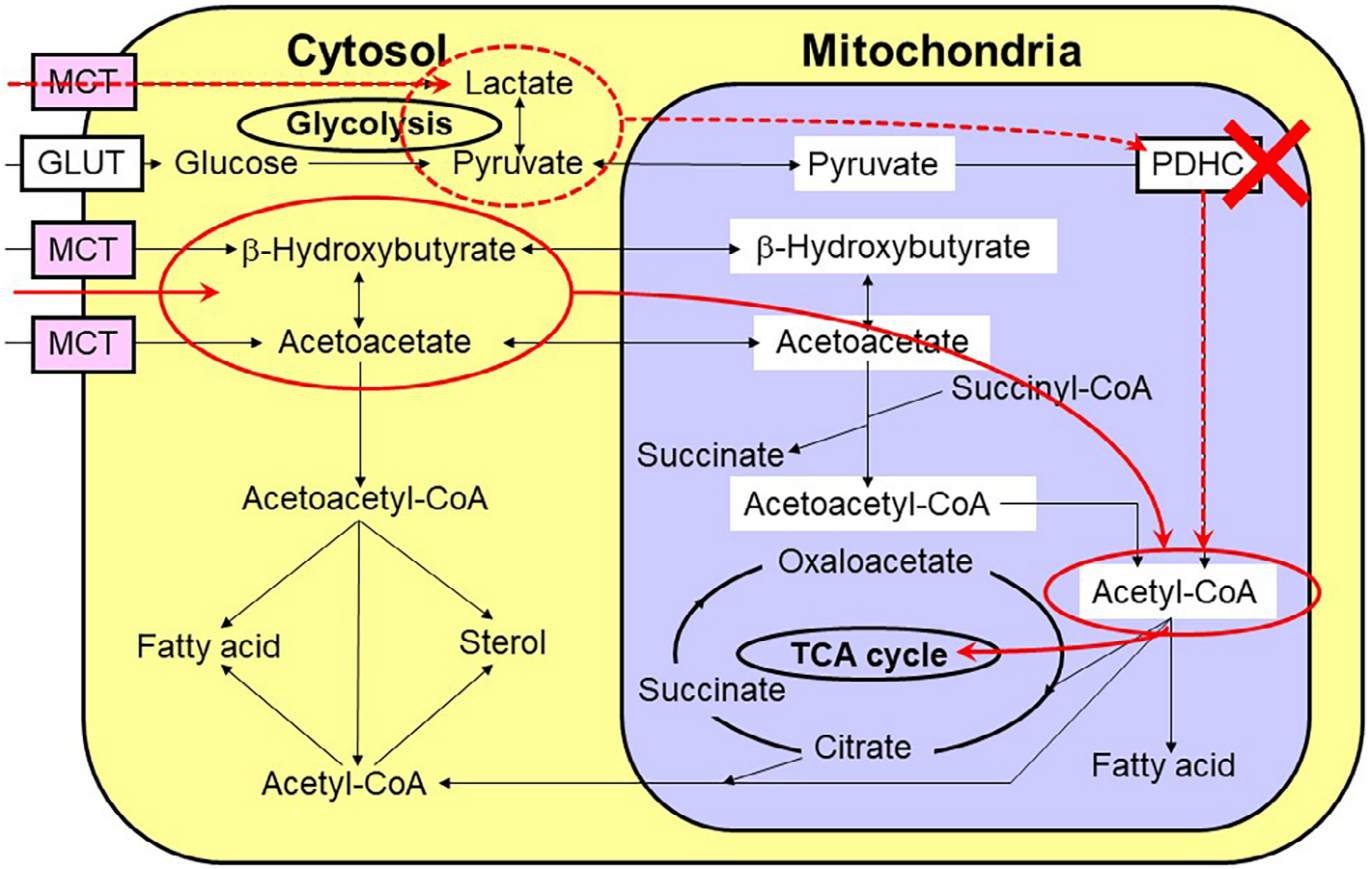
Ketone bodies (KBs) are a better energy source for neurons after ischemia/reperfusion. During ischemia/hypoxia, both lactate and KBs are generated in astroglia. The malfunction of the pyruvate dehydrogenase complex (PDHC) as a result of reperfusion injury enables neurons to utilize KBs, rather than lactate, as a more efficient tricarboxylic acid (TCA) cycle substrate.

Fatty acids and KBs form the second metabolic compartment between neurons and astroglia (Fig. 4).^126^, ^127^, ^128^, ^129^, ^130^, ^131^ KBs, consisting of acetoacetate (AA), acetone, and β‐hydroxybutyrate (BHB), are alternative substrates for glucose. KBs, rather than glucose, are the main energy substrates in the brains of infants. KBs and lactate share the same MCTs. In adults, KBs are produced in the liver under reduced glucose availability, such as starvation and insulin resistance. KBs produced in the liver are transported into the brain where they are utilized by neurons as well as glial cells as substrates for the TCA cycle. After being taken up by neural cells via MCT1 or MCT2, KBs are converted to acetyl‐CoA; a further metabolic process subsequently occurs in the TCA cycle (Fig. 5). In contrast to lactate or pyruvate, which must also be converted to acetyl‐CoA by pyruvate dehydrogenase complex (PDHC) before entering the TCA cycle, KBs do not require PDHC activity. PDHC is a key enzyme in glucose metabolism that links glycolysis and the TCA cycle. Despite being of vital importance, PDHC is susceptible to oxidative stress (Fig. 5).^32^, ^132^, ^133^ Therefore, neurons are not capable of utilizing lactate, which accumulates during cerebral ischemia, after reperfusion (re‐oxygenation) because of PDHC impairment, with ROS being produced in association with the resupply of O_2_ to damaged tissue.

In contrast, KBs, for which PDHC is not required to enter the TCA cycle, can serve as energy substrates for the mitochondrial TCA cycle in neurons after re‐oxygenation. In addition, KBs play neuroprotective roles in several different ways.^32^, ^126^, ^127^, ^128^ More importantly, KBs can be supplied from inside the brain: astroglia are capable of generating KBs and together form a metabolic compartment with neurons (Fig. 4).^32^, ^126^, ^127^, ^128^ KBs are produced from fatty acids as well as an amino acid (leucine).^134^ The main sources of KB production are long‐chain fatty acids. We have evaluated KB production from palmitic acid in astroglia, compared with that in neurons, and our results suggested that KB produced in astroglia can serve as an alternative energy substrate for the TCA cycle in neurons.^32^

### Astroglia produce KBs that act on neurons through transporters and receptors

**Figure 6:**
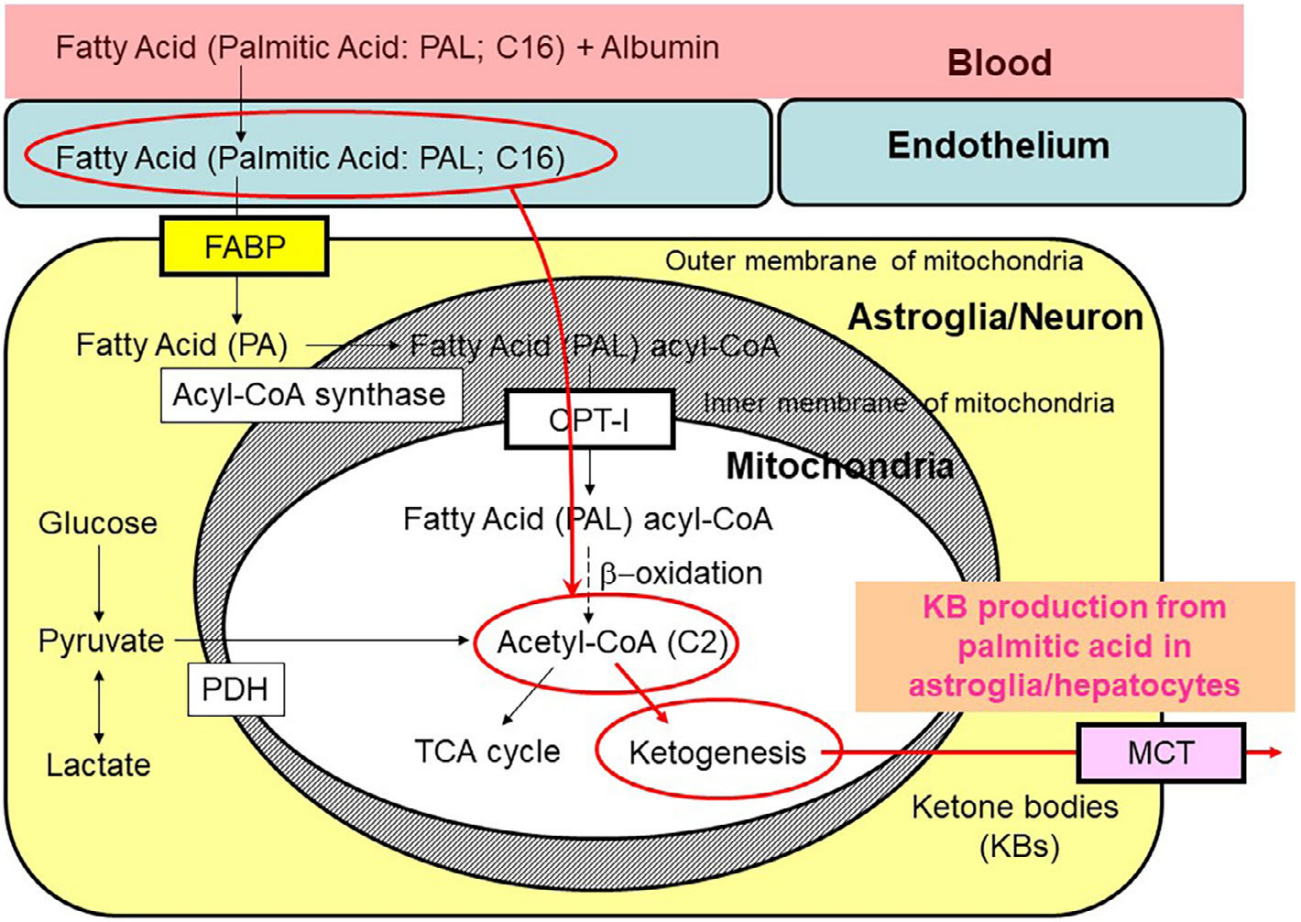
Ketone body (KB) production by astroglia. A long‐chain fatty acid (palmitic acid) is transported into hepatocytes in the liver, generating acetyl‐CoA through β‐oxidation; acetyl‐CoA, in turn, serves as a tricarboxylic acid (TCA) cycle substrate. Astroglia are equipped with a similar metabolic activity and are capable of generating KBs. As KBs are not utilized in the TCA cycle of astroglia, they are transported out through monocarboxylate transporters (MCTs).

Fatty acids are bound to albumin in the blood, and only free fatty acids can cross the BBB.^135^, ^136^ Although the brain levels of soluble fatty acids have not been reported, fatty acids have long been known to be BBB permeable. Neural cells also reportedly express fatty acid‐binding proteins (FABPs) that take up free fatty acids.^137^, ^138^, ^139^, ^140^ Long‐chain fatty acids (> 12 carbons) such as palmitic acid (PAL, 16 carbons), a predominant fatty acid in the body, are then metabolized to produce long‐chain fatty acid acyl‐CoA. Fatty acid acyl‐CoA is then transported into mitochondria by carnitine palmitoyltransferase I (CPT‐I), which exists in the outer membrane of mitochondria and undergoes β‐oxidation. Because astrocytes envelop microvessels in the brain, they are likely to be the main site of fatty acid metabolism in the brain (Fig. 6).^7^

The initial step is β‐oxidation, and we measured KB production using ^14^C‐labeled palmitic acid. Long‐chain fatty acids (e.g., PAL) are an important source for KB production.^132^, ^133^ Although FABP is reportedly expressed in the endothelium and helps with the transportation of fatty acids into the brain, the kinetic properties of the transportation of long‐chain fatty acids into the brain remains a topic of debate. Another source of KB production in the brain is amino acids. Leucine can be converted to KB.^134^

**Figure 7:**
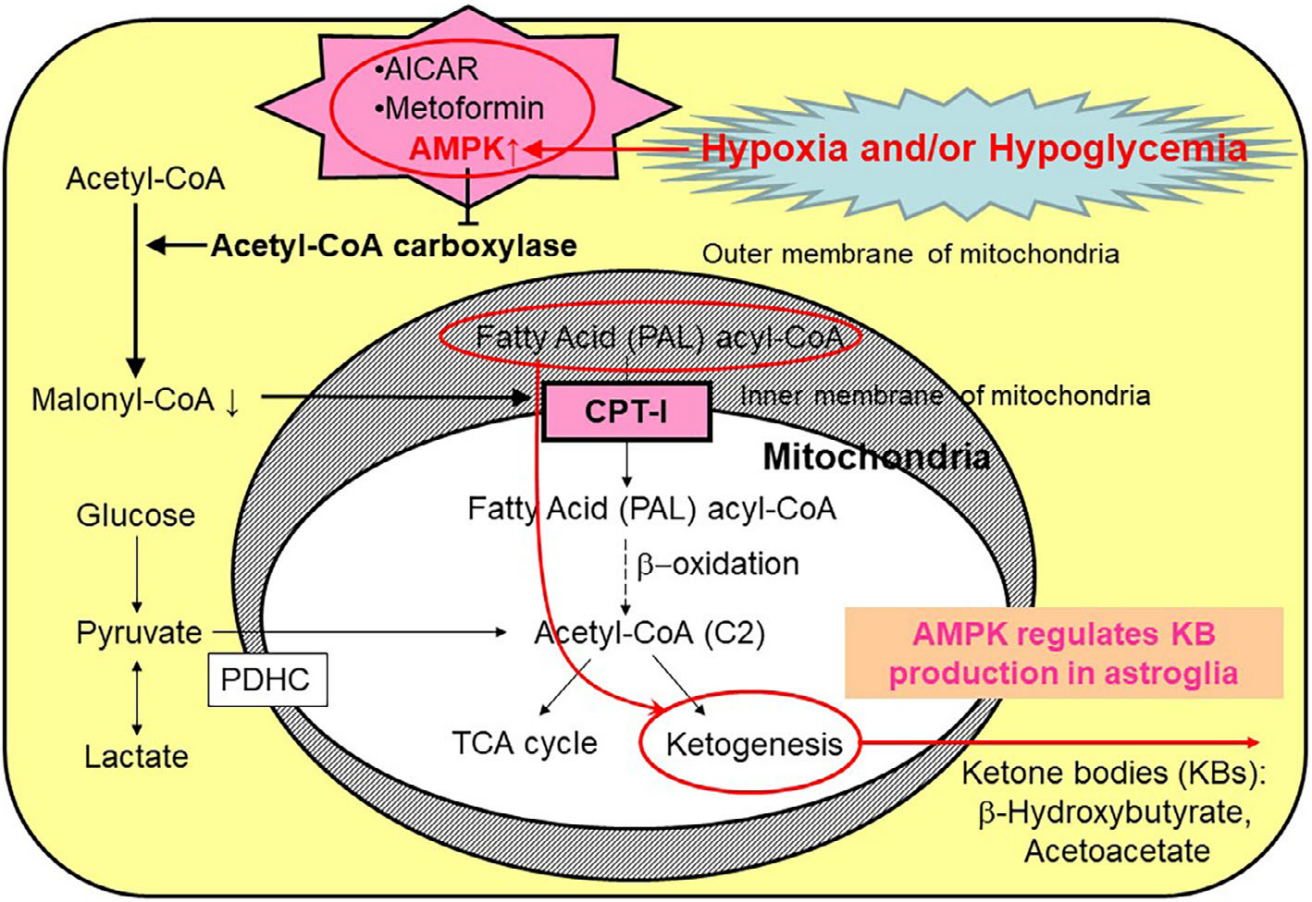
Regulation of ketone body (KB) production by astroglia. Hypoxia and/or hypoglycemia activates adenosine monophosphate‐dependent kinase (AMPK), which phosphorylates and inhibits acetyl‐CoA carboxylase. Decreases in malonyl‐CoA disinhibit carnitine palmitoyltransferase I (CPT‐I), leading to increased KB production. Both 5‐amino‐1‐β‐D‐ribofuranosylimidazole‐4‐carboxamide (AICAR) and metformin activate AMPK and induce increases in KB production in astroglia.

KB production by astroglia is regulated by AMP‐dependent kinase (AMPK), an energy sensor in cells (Fig. 7). When astroglia are exposed to hypoxia and/or hypoglycemia, KB production by astroglia is stimulated by metformin, an oral diabetic drug. Guzmán and Blázquez^126^ reported that AMPK regulates astroglial ketogenesis by phosphorylating acetyl‐CoA carboxylase (ACC), thereby inhibiting ACC activity and reducing cytosolic malonyl‐CoA—a major physiological inhibitor of CPT‐I (a rate‐limiting enzyme of fatty acid metabolism). In fact, 5‐amino‐1‐β‐D‐ribofuranosylimidazole‐4‐carboxamide (AICAR), a cell‐permeable analog of AMP that activates AMPK,^141^ enhances ketogenesis in astroglia. Because AMPK is a sensor of AMP/ATP and acts as an indicator of the energy reserve in cells, Guzmán and Blázquez^126^ speculated that hypoxia/ischemia may stimulate astroglial ketogenesis; they proved that chemical hypoxia induced by 1 mmol/L of NaN_3_, which inhibits cytochrome oxidase and thus the mitochondrial respiratory chain,^142^ for 1 h did indeed enhance ketogenesis in cultured astroglia.^143^

We previously reported that hypoxia and/or hypoglycemia enhances astroglial KB production *in vitro* and that metformin, an AMPK activator, also induces KB production in astroglia (Fig. 7).^32^ Moreover, neurons that had been exposed to hypoxic conditions exhibited reduced oxidative capacities for lactate and pyruvate, while the oxidation of BHB was preserved, suggesting astroglial metabolic support through KB production under ischemia/reperfusion.^32^ Even without the presence of long‐chain fatty acids, astroglia are capable of producing KBs from leucine, although the physiological relevance of this mechanism remains to be determined.

### BHB as a ligand for HCAR2

The recent discovery that BHB acts as an endogenous ligand for hydroxycarboxylic acid receptor 2 (HCAR2) has expanded the role of BHB beyond that of a mere energy substrate.^144^, ^145^, ^146^ An experimental ischemic model showed that the activation of HCAR2 reduced the infarct volume, and this beneficial effect was lost in animals with the genetic deletion of HCAR2.^145^ Interestingly, HCAR2 was expressed mostly in peripheral blood cells. Astroglia‐derived BHB can activate HCAR2 in brain cells more efficiently, and accumulating evidence suggests that microglia in the brain also express HCAR2.^147^

## AMINO ACID AND D‐/L‐SERINE

### Glutamate and NMDA receptor co‐agonists

Glutamate plays a cardinal role in synaptic function through glutamate receptors in postsynaptic neurons. Especially, the *N*‐methyl‐D‐aspartate (NMDA) receptor can act as a double‐edged sword. The NMDA receptor is necessary for normal memory function, and NMDA deficits can induce psychiatric disorders. In contrast, overstimulation of the NMDA receptor induces Ca^2+^ influx and leads to neuronal death, that is, excitotoxicity. These two faces of the glutamate‐NMDA receptor's nature make it difficult to use NMDA antagonists as neuroprotective agents against stroke and neurodegenerative diseases, even though NMDA blockers do exert strong neuroprotection.^148^, ^149^ Another aspect of the NMDA receptor is the discovery of co‐agonists that work in concert with glutamate.^150^, ^151^, ^152^, ^153^, ^154^, ^155^ At present, two different co‐agonists have been identified: D‐serine and glycine. The former acts as a co‐agonist of glutamate in synaptic NMDA receptors, and the latter acts in extra‐synaptic NMDA receptors.^154^

### D‐serine is converted from L‐serine in pre‐synaptic neurons

**Figure 8:**
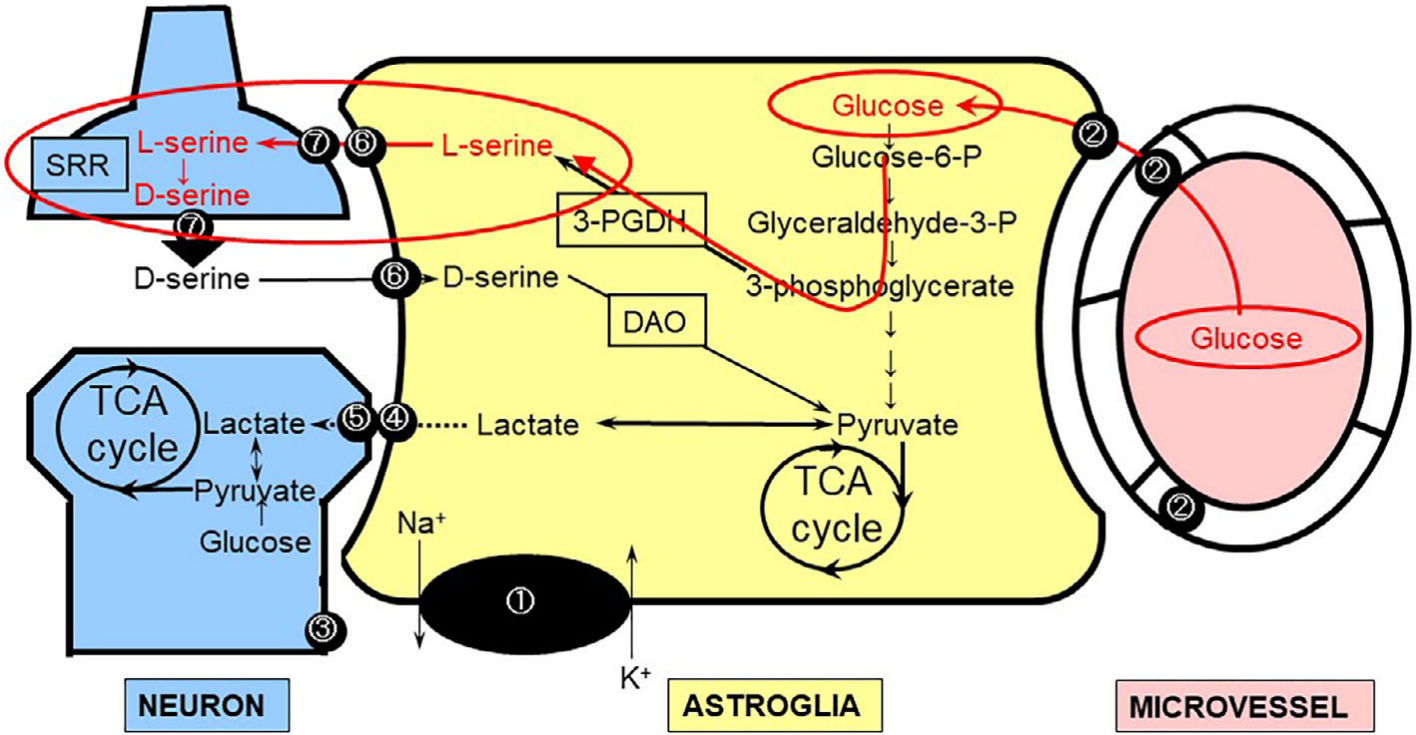
D‐ and L‐serine form amino acid compartmentalization between neurons and astroglia. The *de novo* synthesis of L‐serine occurs in the astroglial glycolytic pathway. L‐serine is transported to neurons, where it is converted to D‐serine by serine racemase (SRR) and acts as a co‐agonist of *N*‐methyl‐D‐aspartate (NMDA) receptors (red line). ① Na^+^,K^+^‐ATPase. ② Glucose transporter 1 (GLUT1). ③ Glucose transporter 3 (GLUT3). ④ Monocarboxylate transporter 1 (MCT1) and MCT4 (astrocytic form). ⑤ MCT2 (neuronal form). ⑥ Astrocytic Na^+^‐independent asc‐type amino acid transporter‐1 (ASCT‐1). ⑦ ASCT‐2.

D‐serine is an enantiomer of L‐serine and is thought to be converted from L‐serine by serine racemase (SRR).^156^, ^157^, ^158^, ^159^ SRR is almost exclusively expressed in pre‐synaptic neurons, indicating that pre‐synaptic neurons release both glutamate and D‐serine into the synaptic cleft and modulate postsynaptic function (Fig. 8).^155^, ^156^ Of note, L‐serine is more abundant in astroglia than in neurons. The *de novo* synthesis of L‐serine occurs exclusively in astroglia, and its synthetic pathway branches at glycolysis, implying the presence of another metabolic compartment between neurons and astroglia (Fig. 8).^157^, ^158^, ^159^

Following the first report of the effect of SRR deletion on ischemic brain damage,^160^ we have evaluated the neuroprotective effect of the elimination of D‐serine in a mouse ischemic stroke model.^161^ SRR‐KO mice exhibited smaller infarct volumes and better functional recovery, compared with control mice, after experimental middle cerebral artery occlusion and reperfusion, indicating that D‐serine deletion can, at least in stroke, be used as a neuroprotective strategy.^161^ However, L‐serine deletion does not necessarily enable neuroprotection.

### De novo synthesis of L‐serine in astroglia and possible neuroprotection

L‐serine is produced in astroglia from the glycolytic pathway via enzyme 3‐phosphoglycerate dehydrogenase (3PGDH).^157^, ^158^, ^159^ The astrocyte‐specific knockout of this enzyme dramatically reduces L‐serine in the brain and probably also reduces D‐serine in neurons. Using the middle cerebral artery occlusion (MCAO) model, the effect of reducing L‐serine on the stroke volume was evaluated. Surprisingly, the infarct volume was not significantly reduced, suggesting an opposite function, that is, neuroprotection, of L‐serine (unpublished data). In fact, Wang *et al*.^162^ found that L‐serine infusion reduced the infarct volume in a mouse MCAO model, and they speculated that this action of L‐serine may be dependent on a vasodilatory effect, since L‐serine increases cerebral blood flow.^163^ Furthermore, a neuro‐restorative role of L‐serine, in addition to its neuroprotective role, has been postulated.^164^

## SUMMARY AND UNSOLVED ISSUES

### Metabolic interaction between astroglia and neurons

Accumulating evidence indicates astroglial metabolic supports through at least three compartments under both normal physiological as well as pathophysiological conditions. The cardinal metabolic compartments are as follows: (i) glucose and lactate; (ii) fatty acids and KBs; and (iii) D‐ and L‐serine. Of note, most available evidence is based on *in vitro* studies using rodent neural cell cultures. A major criticism is that rodent neurons and astroglia might not be an appropriate model for human brain cells.^165^ In particular, the high glycolytic metabolic activity with lactate production might be a characteristic of rodent cell cultures only. Recent technology utilizing induced‐pluripotent stem cells (iPSCs) has enabled us to evaluate human neurons and astroglia *in vitro*. Thus far, only a limited number of studies have focused on the metabolic compartments between these types of cells.^166^ We recently induced cortical astroglia and spinal motor neurons from iPSCs prepared from normal healthy adults and measured the glycolytic activities of both cell types. So far, the astroglial glycolytic capacity seems to be higher than the neuronal capacity (unpublished data). Further confirmation of these findings using *in vivo* human brain studies is warranted.

### Compartmentalization between oligodendroglia and astroglia

In the white matter of the brain, the axons of neurons are myelinated by oligodendrocytes, which enable saltatory conduction. Similar to the gray matter of the brain, small vessels are completely covered by astroglial end‐feet.^7^ Thus, substrates for energy production as well as structure construction must be supplied through the astroglia to the oligodendroglia. More precisely, at Ranvier nodes, where the ionic flux is most active, the astroglial end‐feet are in direct contact with axons.^167^, ^168^ Assuming that axons utilize lactate preferentially as an energy substrate for the TCA cycle, how lactate is supplied to the axons is an interesting and unsolved issue: is lactate supplied by oligodendroglia or astroglia? Astroglia can supply lactate directly at Ranvier nodes, similar to their actions at tripartite synapses. However, a recent report has elucidated that oligodendroglia metabolize glucose glycolytically to produce lactate.^169^, ^170^ Glucose supplied from microvessels can be used as a direct substrate for oligodendroglia to support neuronal energy metabolism.^9^, ^171^, ^172^ The CMR_glc_ in the white matter is much less than that in the gray matter, and the exact contributions of astroglia, oligodendroglia, and neurons (axons) to CMR_glc_ and the pathways of glucose transport to oligodendroglia and neurons remain to be solved.

The fate of KBs produced in astroglia in the white matter should be evaluated from the perspective of myelin formation. KBs can be utilized in lipid synthesis for the cell membrane.^173^, ^174^, ^175^ Membrane formation is dependent on cholesterol, which can be supplied directly from astroglia as KBs. A dysfunction in cholesterol trafficking has been implicated in memory impairment.^175^ How astroglial metabolic support is needed for myelination by oligodendroglia remains to be elucidated.^176^

### Energy metabolism in microglia

Microglia are the last type of glial cells in the brain. Although the origin of this type of glial cells seems to differ from that of other glial cells, microglia reside in the brain during the early developmental stage and play a pivotal role in the immunological response of the brain by regulating synaptic pruning and scavenging damaged neurons.^177^, ^178^ As mentioned in previous chapters, microglia have a strong influence on astroglial responses of either a harmful or beneficial nature.^33^, ^125^ We have examined the interaction between astroglia and microglia via NO. Upon stimulation by LPS, a classical ligand of TLR4, microglia are activated to produce NO which, in turn, induces neuroprotective astroglia through the Keap1/Nrf2 system.^33^ Moreover, LPS stimulation activates NADPH oxidase (NOX), which is highly expressed in microglia. In fact, microglia have a strong capacity to produce ROS and, therefore, the anti‐oxidative system should also be equipped to protect itself. A limited number of studies have revealed that the microglial PPP is active probably to keep the glutathione system active, as in astroglia. Our observations suggest that the microglial PPP flux is as high as the astroglial one (unpublished data), suggesting a high glycolytic metabolism in microglia.

Another issue to be solved is how microglial energy production is regulated in the light of physiological and pathophysiological aspects.^179^, ^180^ Namely, microglia can be ameboid in shape and can travel upon various stimulations. The physical movement of cells requires more energy than that of cells in a quiescent state. Whether microglia utilize glucose in the extracellular space or lactate or KBs is an interesting question that remains to be answered. Fatty acids could be another candidate, as cardiac and skeletal muscles preferentially utilize fatty acids over glucose. The alteration of MCT expression in microglia has been reported, indicating that lactate or KBs may also be energy substrates for microglial energy metabolism.^181^

## DISCLOSURE

The author declares no conflict of interest.
